# Minimal Hepatic Encephalopathy is Associated with Increased Cerebral Vascular Resistance. A Transcranial Doppler Ultrasound Study

**DOI:** 10.1038/s41598-019-51867-6

**Published:** 2019-10-25

**Authors:** Francesca Romana Ponziani, Barbara Funaro, Andrea Lupascu, Maria Elena Ainora, Matteo Garcovich, Gianluigi Caracciolo, Alessandro Quadarella, Antonio Nesci, Laura Riccardi, Antonio Gasbarrini, Maurizio Pompili, Maria Assunta Zocco

**Affiliations:** 1 Internal Medicine, Gastroenterology and Hepatology, Fondazione Policlinico Agostino Gemelli IRCCS, Rome, Italy; 2 Internal Medicine, Fondazione Policlinico Agostino Gemelli IRCCS, Rome, Italy

**Keywords:** Liver cirrhosis, Hepatic encephalopathy

## Abstract

Minimal hepatic encephalopathy (MHE) is a subclinical complication of liver cirrhosis with a relevant social impact. Thus, there is urgent need to implement easy to use diagnostic tools for the early identification of affected patients. The aim of this study was to investigate cerebral blood flow, systemic hemodynamics as well as endothelial function of cirrhotic patients with MHE, and to verify their change after treatment with rifaximin. Fifty cirrhotic patients with or without MHE and an equal number of healthy controls underwent transcranial Doppler ultrasound (TCD), abdominal Doppler ultrasound (US), and measurement of flow mediated dilation (FMD). In cirrhotic patients diagnosed with MHE receiving rifaximin, the tests were repeated at the end of treatment. Middle (MCA) and posterior (PCA) cerebral artery resistive (RI) and pulsatility (PI) indices were higher in cirrhotic patients than controls, as well as renal and splenic artery RI. Conversely, FMD was reduced. MCA-RI and PI were even higher in cirrhotic patients with MHE compared to those without; a MCA-RI cut-off of 0.65 showed an accuracy of 74% in discriminating the presence of MHE, with 65% sensitivity and 76% specificity. Rifaximin treatment showed no efficacy in the modulation of cerebral vascular flow. In conclusion, cirrhotic patients with MHE have significantly increased cerebral vascular resistances that are not improved by rifaximin treatment. MCA-RI measurement has a good accuracy for the diagnosis of MHE and can be useful for the early identification of this harmful complication of liver cirrhosis.

## Introduction

Minimal hepatic encephalopathy (MHE), which affects up to 70% of patients with liver cirrhosis^1^, represents a heavy medical and social burden, being associated with worse survival, reduction in the quality of life, work capacity and ability to drive^1–5^. Thus, it is crucial to identify which patient presents with MHE, in order to implement therapeutic measures that can avoid its harmful consequences.

MHE does not imply clear clinical manifestations, so it can be diagnosed only by specific tests. Neuropsychological evaluation is the main tool in the detection and quantification of MHE, as the most altered cognitive domains are well characterized and mainly concern psychomotor speed and accuracy, attention and executive functions. The international guidelines agree in recommending the use of paper pencil or computerized psychometric or psychophysiological tests for the diagnosis of MHE in clinical practice, after having ruled out other causes of brain function impairment^6,7^.

The indication for the treatment of MHE is not univocal, rather it is to be decided on a case-by-case basis, and includes dietary measures, the use of non-absorbable disaccharides and non-absorbable antibiotics (mainly rifaximin)^6,7^. Furthermore, parameters for monitoring the effectiveness of medical therapy in patients with MHE are not currently coded, but should take into account cognitive performance and daily life autonomy^6,7^.

The alteration of cerebral vascular flow has been reported in patients with hepatic encephalopathy, but it is unclear whether this may be the cause or the consequence of the encephalopathy itself^8–12^. Studies are mainly based on nuclear radiology imaging techniques, such as SPECT, PET or Xenon scintigraphy, which are expensive and often not available in smaller centers; there are few published data on the assessment of the cerebral vascular flow by transcranial Doppler ultrasound (TCD) in patients with liver cirrhosis and, among them, in those with hepatic encephalopathy, describing an increase in the resistive and pulsatility indices (RI and PI, respectively)^10,13,14^.

However, only one study included patients with MHE, but it was not specifically focused on it^13^. Nevertheless, at present, there are no data concerning the variation of cerebral hemodynamics after MHE treatment with rifaximin or the correlation between cerebral and systemic flow parameters and endothelial function in patients with MHE.

In light of the above, the primary aims of this study were a) to evaluate if there were changes in RI and / or PI measured by TCD in the cerebral arteries of cirrhotic patients with or without MHE; b) to evaluate the effects of rifaximin treatment on the cerebral hemodynamic parameters. As secondary endpoints, we investigated the differences in splanchnic hemodynamics and endothelial function in cirrhotic patients with MHE compared to those without, their correlation with cerebral vascular resistance, and whether they can be changed by rifaximin therapy.

## Methods

All cirrhotic patients to be included in the study were evaluated at the Department of Internal Medicine, Gastroenterology and Hepatology of the Fondazione Policlinico A. Gemelli in Rome. The protocol was approved by the Ethics Committee of our Institution (ID 1712) and carried out in accordance with relevant guidelines and regulations (NCT04077125; registered on September 4, 2019). The informed consent was obtained from all subjects.

The diagnosis of liver cirrhosis was made on the basis of clinical, laboratory and ultrasound findings. Clinically significant portal hypertension was diagnosed in patients with splenomegaly and decreased platelet count, and/or ascites, and/or gastric or esophageal varices. The severity of liver disease was assessed according to the Child-Pugh classification. Healthy subjects chosen among the medical staff and matched by age were also included in the study as control group.

The following exclusion criteria have been adopted for all the study population: age <18 years; active alcohol abuse (excessive alcohol intake stopped more than 6 months before the enrollment); chronic pulmonary diseases; ongoing infections; cerebrovascular diseases; primary or secondary cerebral neoplasm; primary liver neoplasm; heart function failure; chronic kidney disease; peripheral vascular disease; treatment with rifaximin or systemic antibiotics in the previous 15 days; smoking habit; grade 1 or overt hepatic encephalopathy.

In the first part of the study, all cirrhotic patients underwent:

- neuropsychological tests such as the trail making test (TMT) A and B and the digit symbol test (DST);
- TCD with measurement of RI and PI of the right middle cerebral artery (MCA) and posterior cerebral artery (PCA);
- flow mediated dilation (FMD) as endothelial function test;
- abdominal ultrasound (US) with Doppler measurement of the mean portal flow velocity (MPV), and of the renal and splenic arteries RI (RA-RI and SA-RI, respectively);
- blood assay for the assessment of serum ammonia and of total bilirubin, INR and albumin levels for the calculation of the Child-Pugh score.

The same examinations were performed in the control group, except for the neuropsychological tests and laboratory examinations. The operators performing TCD, FMD and abdominal US were unaware of the results of the neuropsychological tests. Based on the presence or absence of abnormal results, cirrhotic patients were then divided into two groups: patients with MHE (MHE group) and patients without MHE (no MHE group).

In the second part of the study, patients in the MHE group were treated with rifaximin (400 mg tid orally) for 15 days on a case by case basis, mainly taking into account the impairment of the quality of life. At the end of the treatment period, the same tests and examinations performed at the baseline were repeated.

### Neuropsychological tests

The diagnosis of MHE was made using the paper and pencil tests. According to the present international guidelines, in the clinical routine or in single-center studies investigators should use tests with which they are familiar and for which normative reference data are available^6,7^.

At the time when the study was designed, the TMTA, TMTB and DST^15^ were the only neuropsychological tests validated for the Italian population and were therefore adopted. The tests were performed by a skilled clinician with 10 years of experience in the use of the tests (F.D.); the results obtained were appropriately corrected for age and educational level and expressed as differences (in standard deviation) compared to reference values (Z-scores); thus, the diagnosis of MHE was made when at least one test was impaired beyond 2 standard deviations compared to control values (Z-score <2).

### TCD

The TCD was performed with the Affinity 70 C Ultrasound System (Philips, Amsterdam, The Netherlands) equipped with 5-1 MHz linear sector probe. The right MCA and PCA were identified by US and color Doppler study and the sampling volume of the pulsed Doppler was placed in the arteries at a depth of 45–60 mm from the ultrasound emission point^16^. The peak systolic velocity, the mean velocity and the minimum telediastolic velocity were determined. The RI and PI were automatically calculated according to the following formulas:

- PI = (peak systolic velocity – minimum telediastolic velocity)/mean velocity;
- RI = (peak systolic velocity – minimum telediastolic velocity)/peak systolic velocity.

### Abdominal Doppler US examination

The abdominal Doppler US was performed on the same day of TCD with a IU22 US system (Philips, Amsterdam, The Netherlands) equipped with a wideband C5-2 MHz convex probe by two experienced sonographers (L.R. and M.A.Z. with 18 and 12 years of experience in abdominal US respectively). Each patient was placed in the supine position and fasted for at least 6 hours to avoid any influence of posture and ingested food on the splanchnic hemodynamics. All Doppler US parameters were obtained during suspended respiration^17^.

Among different parameters, reported to be altered in case of cirrhosis and portal hypertension and currently used in clinical practice, MPV, RA-RI and SA-RI were analyzed. The mean of three consecutive measurements was calculated for each parameter.

Time-averaged mean maximum velocity of the portal vein was measured in centimeters per second by placing the Doppler sample volume, with a width of approximately half of the lumen, in the middle of the portal trunk using a Doppler angle of less than or equal to 60° for angle correction^18^.

For the evaluation of SA-RI, the transducer was held below the left costal margin or in the left costal spaces. The sample volume of the Doppler system was placed in the main parenchymal branches of the splenic artery and the blood flow velocity waveform was recorded for peak systolic and telediastolic flow velocity evaluation and RI calculation, without correction for the angle of insonation^19^.

After US demonstration of normal morphology of the kidneys, the RA-RI was measured in both kidneys by placing the sample volume in the interolobar arteries.

Peak systolic and telediastolic flow velocity were then determined without correction for the angle of insonation, and the RI was calculated as previously reported. In the absence of differences exceeding 0.05, the mean RI of the right kidney was used; otherwise the highest value was chosen.

### Endothelial function

The endothelial function was determined by the brachial artery FMD test. The examination was performed with the patient under fasting conditions, at the same time of day (range 8–9 am), avoiding substances or situations potentially affecting the hemodynamic parameters such as caffeine, tobacco, alcohol, physical exercise during the previous 12 hours. The subjects were placed in a supine position in a quiet room for 15 minutes before the test. A standard cuff was placed around the right arm, about 4 cm below the antecubital fossa. A linear 12-3 MHz probe was connected to the iU22 (Philips) ultrasound device to identify the right brachial artery. After the acquisition of the baseline images, the cuff was inflated to above 250 mmHg to occlude the right brachial artery, and kept inflated for 5 minutes. This caused a transient ischemia, inducing the vasodilation of resistance vessels through self-regulation mechanisms, and reaching a peak approximately 60 seconds after deflating the cuff. The maximum increase in the brachial artery diameter was then recorded and FMD was expressed as percentage of brachial artery dilation compared to baseline.

### Statistical analysis

Statistical analysis was performed using non-parametric tests based on the non-normal distribution of data. Continuous and ordinal variables were expressed as median and interquartile (IQR) range, categorical variables as frequencies and percentages.

Baseline differences in the cerebral and abdominal arteries flow parameters, FMD, ammonia serum level, neuropsychological tests results, age, sex, etiology of liver disease and Child-Pugh score between cirrhotic patients and healthy controls, then between cirrhotic patients with MHE and those without were assessed using either the Wilcoxon test for independent samples or the Fisher’s exact test; variables presenting a significant association with MHE were included in a logistic regression model. Significant correlations between the cerebral and abdominal arteries hemodynamic parameters, FMD, and ammonia serum level were investigated using Spearman’s correlation.

A Receiver Operating Characteristic (ROC) curve was plotted to assess the diagnostic performance of cerebral arteries RI and PI cut-points in distinguishing cirrhotic patients with MHE from those without.

Finally, treatment-related differences in cerebral and splanchnic vascular resistance, FMD and ammonia serum level in patients with MHE who underwent rifaximin therapy were evaluated using the Wilcoxon test for paired data.

All statistical significance tests used were two-tailed and a p < 0.05 indicates a statistically significant difference. Statistical analysis was performed using software R version 3.4.0.

## Results

### Characteristics of the study population

A total of 77 cirrhotic patients were evaluated, but 27 of them were not included in the analysis due to one or more exclusion criteria (11 had clinical signs of overt hepatic encephalopathy, 5 were treated with rifaximin, 7 were smokers, 3 refused to participate in the study and 1 was affected by peripheral artery disease). Thus, 50 cirrhotic patients and an equal number of controls were enrolled in the study (Table 1).

**Table 1:** Clinical and demographic characteristics of the cirrhotic patients and healthy controls included in the study.

VARIABLE	MEDIAN (IQR)/COUNT (%) CONTROLS (50)	MEDIAN (IQR)/COUNT (%) CIRRHOSIS (noMHE + MHE) (50)	p-value CIRRHOSIS vs. CONTROLS	MEDIAN (IQR)/COUNT (%) CIRRHOSIS no MHE (30)	MEDIAN (IQR)/COUNT (%) CIRRHOSIS MHE (20)	p-value CIRRHOSIS MHE vs. no MHE
*Age (years)*	58 (49–66)	61 (53–66)	0.576	61 (55–66)	58 (42–67)	0.393
*Sex*						
*male*	26 (52)	33 (60)	0.222	21 (70)	12 (60)	0.548
*female*	24 (48)	17 (40)		9 (30)	8 (40)	
*Etiology*-						
*viral*	—	26 (52)		16 (53)	10 (50)	0.999
*non viral*		24 (48)		14 (47)	10 (50)	
*Child*-*Pugh*	—		—			
-*A*		32 (64)		23 (76.7)	9 (45)	0.054
-*B*		13 (26)		5 (16.6)	8 (40)	
-*C*		5 (10)		2 (6.7)	3 (15)	
*MCA*-*RI*	0.54 (0.53–0.58)	0.63 (0.61–0.68)	**<0.0001**	0.61 (0.57–0.64)	0.67 (0.62–0.72)	**0.004**
*MCA*-*PI*	0.84 (0.82–0.93)	1.10 (1.03–1.24)	**<0.0001**	1.05 (0.94–1.13)	1.16 (1.07–1.36)	**0.012**
*PCA*-*RI*	0.57 (0.52–0.62)	0.61 (0.56–0.67)	**0.004**	0.61 (0.55–0.66)	0.64 (0.58–0.68)	0.305
*PCA*-*PI*	0.91 (0.84–1.03)	1.02 (0.89–1.23)	**0.005**	1 (0.87–1.17)	1.08 (0.95–1.29)	0.300
*RA*-*RI*	0.58 (0.57–0.60)	0.66 (0.61–0.70)	**<0.0001**	0.67 (0.61–0.71)	0.64 (0.60–0.69)	0.306
*SA*-*RI*	0.50 (0.49–0.51)	0.64 (0.61–0.71)	**<0.0001**	0.69 (0.61–0.72)	0.63 (0.60–0.65)	0.084
*MPV (cm/s)*	26.80 (25.82–27.47)	23.75 (19.30–27.10)	**0.0006**	22 (18–25.5)	27 (23.55–29)	**0.008**
*FMD (%)*	10.69 (7.78–13.24)	6.35 (3.40–9.68)	**0.0003**	6.44 (5.20–9.71)	5.35 (1.18–10.20)	0.405
*Ammonia (mcg/dL)*	—	75 (58–122)	—	67 (44.5–90.50)	114 (79.5–141.25)	**0.010**
*TMTA (sec)*	—	45 (34.5–61.5)	—	42.5 (29.75–54.25)	59 (38.50–87.50)	**0.019**
*TMTB (sec)*	—	89 (65–27)	—	72.5 (56.5–104.75)	95 (81–182)	**0.008**
*DST (items)*	—	26 (19.5–34)	—	30 (25–39.25)	18 (14–24)	**<0.0001**
*TMTA zeta*	—	−0.245 (−1.015–0.567)	—	0.205 (−0.545–0.79)	−0.98 (−1.555 – −0.067)	**0.007**
*TMTB zeta*	—	0.13 (−1.13–0.75)	—	0.45 (−0.137–1.225)	−0.325 (−1.75–0.235)	**0.001**
*DST zeta*	—	−1.3 (−2.105 – −0.775)	—	−0.9 (−1.24–0.22)	−2.205 (−2.64 −2.03)	**<0.0001**

RI = resistive index; PI = pulsatility index; MCA = middle cerebral artery; PCA = posterior cerebral artery; RA = renal artery; SA = splenic artery; MPV = mean portal vein velocity; FMD = flow mediated dilation; TMT = trail making test; DST = digit symbol test.

Cirrhotic patients had a median age of 61 years (range 53–66), 33 were male and 17 female. In 52% of them the etiology of liver disease was related to chronic hepatitis B or C virus infection, whereas in 48% it was related to previous excessive alcohol intake or non alcoholic fatty liver disease. Most of the patients were classified as Child-Pugh A (64%), 26% as Child-Pugh B and 10% as Child-Pugh C.

After the neurophsycological assessment, 20 out of the 50 cirrhotic patients were diagnosed with MHE. Their median age was 58 years (range 42–67), with a prevalence of men (60%). Forty-five percent of them were classified as Child Pugh A, 40% as Child-Pugh B and 15% as Child-Pugh C, with a comparable prevalence of viral or non-viral etiology of liver disease. There were no significant differences in age, sex, etiology of the disease and liver function assessment between cirrhotic patients with or without MHE. Only ammonia serum level was significantly higher in patients with MHE [114 (79.5–141.25) vs. 67 (44.5–90.50) mcg/dL, p = 0.010].

### Cerebral and systemic hemodynamics of cirrhotic patients

Table 1 shows the comparisons between cerebral hemodynamic parameters (MCA-RI and MCA-PI, PCA-RI and PCA-PI), RA-RI, SA-RI, MPV, and FMD between patients and controls, and between patients with or without MHE. Due to technical limitations, PCA-RI and PCA-PI could not be measured in two patients.

MCA-RI and MCA-PI were higher in cirrhotic patients than controls [MCA-RI: 0.63 (0.61–0.68) vs. 0.54 (0.53–0.58), p < 0.0001; MCA-PI: 1.10 (1.03–1.24) vs. 0.84 (0.82–0.93), p < 0.0001]; similar differences were observed for the PCA-RI and PCA-PI [PCA-RI: 0.61 (0.56–0.67) vs. 0.57 (0.52–0.62), p = 0.004; PCA-PI: 1.02 (0.89–1.23) vs. 0.91 (0.84–1.03), p = 0.005].

Compared to controls, cirrhotic patients also showed an increased RA-RI [0.66 (0.61–0.70) vs. 0.58 (0.57–0.60), p < 0.0001] and SA-RI [0.64 (0.61–0.71) vs. 0.50 (0.49–0.51), p < 0.0001], and lower values of MPV [23.75 (19.3–27.1) vs. 26.80 (25.82–27.47) cm/sec, p = 0.0006] and FMD [6.35 (3.40–9.68) vs. 10.69 (7.78–13.24) %, p = 0.0003].

**Figure 1:**
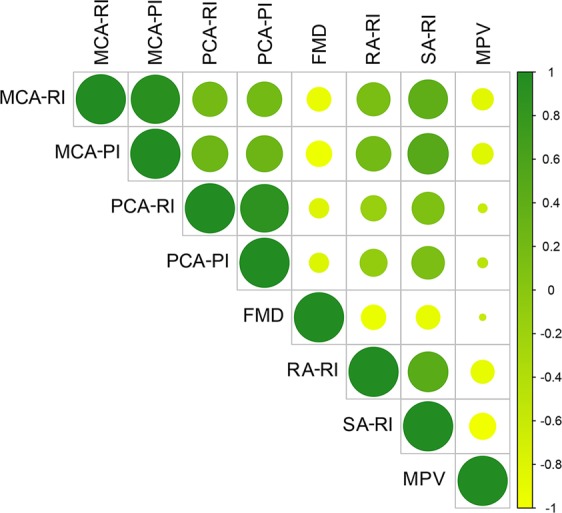
Correlations between systemic and splanchnic vascular resistance parameters and flow mediated dilation (FMD) in the study population. Positive correlations are represented in dark green, negative ones in light green. The size of the circle and color intensity are proportional to the correlation coefficient. RI = resistive index; PI = pulsatility index; MCA = middle cerebral artery; PCA = posterior cerebral artery; RA = renal artery; SA = splenic artery; MPV = mean portal vein velocity; FMD = flow mediated dilation.

Interestingly, cerebral vascular resistance indices were correlated with the splanchnic hemodynamic parameters (Fig. 1). Indeed, we observed a direct correlation between RA-RI and MCA-RI (r = 0.451, p < 0.0001), MCA-PI (r = 0.493, p < 0.0001), PCA-RI (r = 0.263, p = 0.010) and PCA-PI (r = 0.301, p = 0.032); likewise, SA-RI was directly correlated with MCA-RI (r = 0.620, p < 0.0001), MCA-PI (r = 0.674, p < 0.0001), PCA-RI (r = 0.422, p < 0.0001) and PCA-PI (r = 0.438, p < 0.0001). In contrast, FMD showed an inverse association with MCA-RI (r = −0.234, p = 0.018) and MCA-PI (r = −0.268, p = 0.006). No significant correlation between age or ammonia serum level and the cerebral vascular resistance parameters was observed. Furthermore, in patients on treatment with beta-blockers (31) or aldosterone antagonists (22) no difference in cerebral hemodynamic parameters was observed.

As regards cirrhotic patients with MHE, they presented higher MCA-RI [0.67 (0.62–0.72) vs. 0.61 (0.57–0.64) p = 0.004], MCA-PI [1.16 (1.07–1.36) vs. 1.05 (0.94–1.13), p = 0.012] and MPV [27 (23.55–29) vs. 22 (18–25.5) cm/sec, p = 0.008] compared to those without MHE. Conversely, there was no difference in PCA-RI and PCA-PI [PCA-RI: 0.64 (0.58–0.68) vs. 0.61 (0.55–0.66), p = 0.305; PCA-PI: 1.08 (0.95–1.29) vs. 1.00 (0.87–1.17), p = 0.300], FMD [5.35 (1.18–10.20) vs. 6.44 (5.20–9.71) %, p = 0.405], RA-RI [0.64 (0.60–0.69) vs.0.67 (0.61–0.71), p = 0.306] and SA-RI [0.63 (0.60–0.65) vs. 0.69 (0.61–0.72), p = 0.084]. In the binomial regression model, MCA-RI was the only independent variable predictive of MHE [OR 1.53 95%CI (1.23, 1.78), p = 0.022].

**Figure 2:**
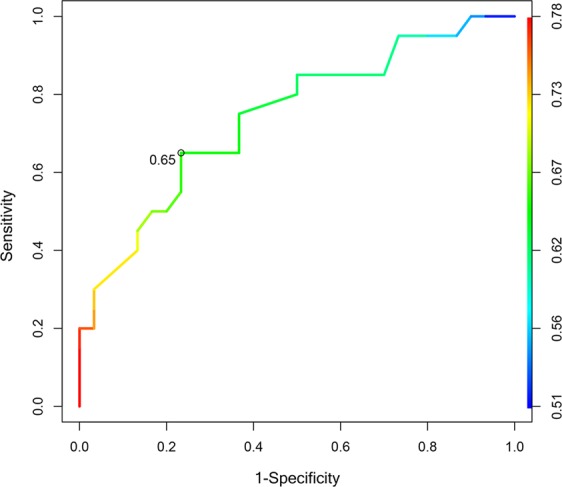
Receiver operating characteristic (ROC) curve of middle cerebral artery resistive index (MCA-RI) for discriminating the presence or the absence of minimal hepatic encephalopathy (MHE). The cut-off value of 0.65 resulted in the best accuracy (74%), with 65% sensitivity and 76% specificity.

In particular, MCA-RI cut-off of 0.65 showed an accuracy of 74% in discriminating cirrhotic patients with MHE from those without, with 65% sensitivity and 76% specificity (Fig. 2); in this setting, the MCA-PI performance was worse (71% accuracy, 60% sensitivity and 76% specificity for a cut-off of 1.15).

### Effect of rifaximin on cerebral vascular resistance and endothelial function in patients with MHE

**Figure 3:**
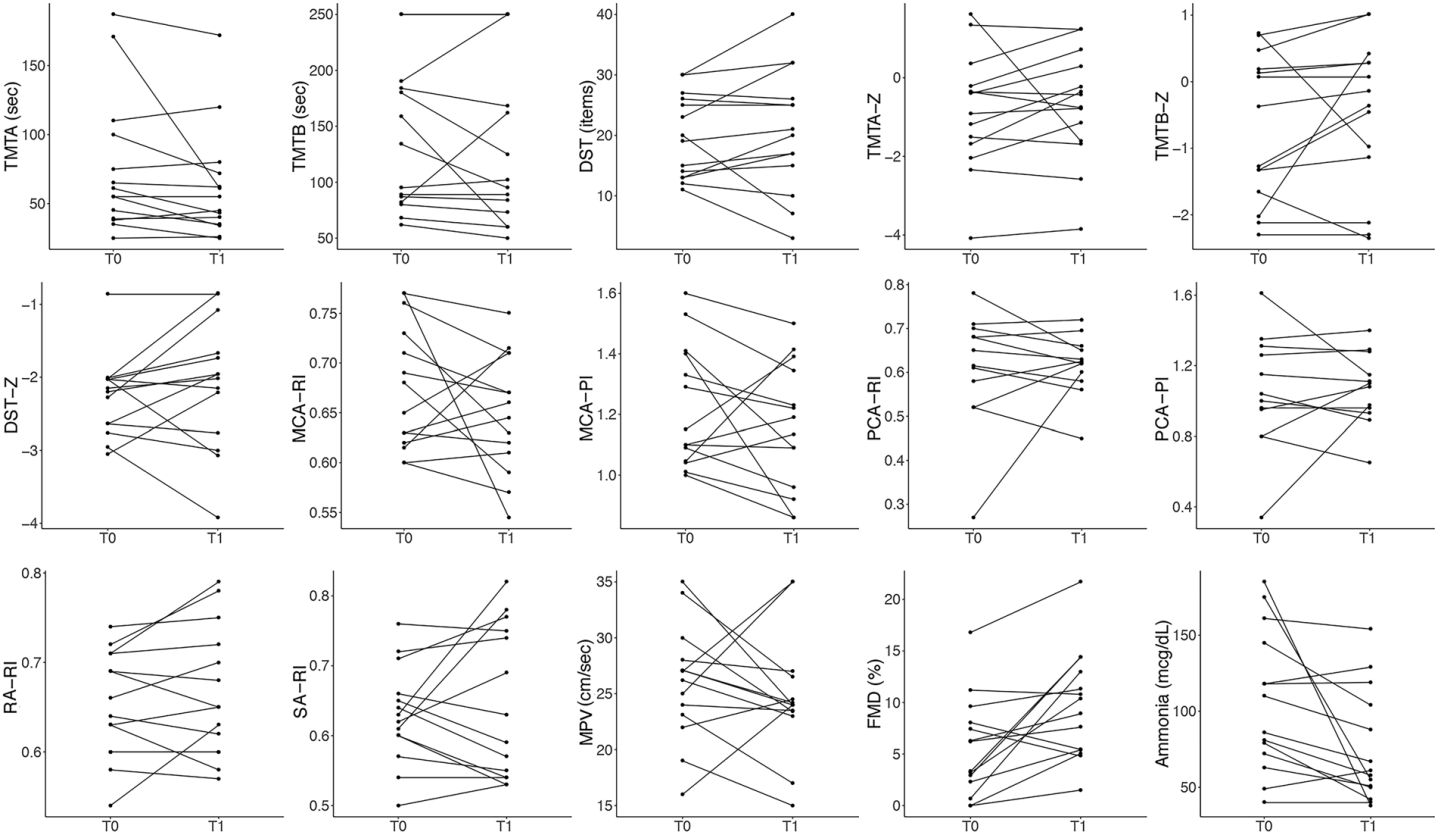
Change in cerebral and splanchnic vascular resistance parameters, flow mediated dilation (FMD), neuropsychological tests results (expressed as rough or standardized values) and ammonia serum level after rifaximin treatment in cirrhotic patients with minimal hepatic encephalopathy (MHE). RI = resistive index; PI = pulsatility index; MCA = middle cerebral artery; PCA = posterior cerebral artery; RA = renal artery; SA = splenic artery; MPV = mean portal vein velocity; FMD = flow mediated dilation; TMT = trail making test; DST = digit symbol test.

Among the 20 cirrhotic patients with MHE, 17 were treated with rifaximin at the dose of 400 mg tid for 15 days. Post-treatment (T1) neuropsychological and hemodynamic parameters were then compared to those recorded at the baseline evaluation (T0) (Fig. 3 and Table 2).

**Table 2:** Variation of cerebral and splanchnic vascular parameters, endothelial function, ammonia serum level and neuropsychological tests in patients who received rifaximin treatment. Values are expressed as median (IQR)/count (%).

VARIABLE	CIRRHOSIS MHE T0 (17)	CIRRHOSIS MHE T1 (17)	p-value
*MCA*-*RI*	0.66 (0.62–0.72)	0.65 (0.61–0.70)	0.299
*MCA*-*PI*	1.12 (1.05–1.38)	1.16 (0.99–1.31)	0.209
*PCA*-*RI*	0.63 (0.56–0.68)	0.62 (0.59–0.65)	0.569
*PCA*-*PI*	1.02 (0.91–1.27)	1.09 (0.95–1.17)	0.929
*RA*-*RI*	0.65 (0.60–0.70)	0.65 (0.60–0.71)	0.325
*SA*-*RI*	0.62 (0.60–0.65)	0.61 (0.54–0.74)	0.806
*MPV (cm/s)*	26.6 (23.32–27.77)	24 (23.42–26)	0.414
*FMD (%)*	4.76 (2.45–7.89)	9.65 (5.41–12.57)	**0.015**
*Ammonia (mcg/dL)*	98 (73.75–138.25)	59.5 (50.25–100)	**0.010**
*TMTA* (*sec)*	58 (40.50–93.75)	50 (36.25–69.50)	0.068
*TMTB (sec)*	114.5 (83.25–183)	98.5 (75.75–166.5)	0.327
*DST (number)*	19.5 (13.25–25.75)	20.5 (15.50–25.75)	0.380
*TMTA zeta*	−0.65 (−1.645–0.247)	−0.59 (−1.495–0.160)	0.267
*TMTB zeta*	−0.82 (−1.577–0.175)	−0.25 (−1.100–0.208)	0.168
*DST zeta*	−2.17 (−2.640–2.030)	−1.99 (−2.622 −1.687)	0.248

The results of the TMTA, TMTB and DST tests slightly improved, but the cerebral and abdominal vascular resistance indices remained stable. Conversely, there was a significant increase in FMD value [9.65 (5.41–12.57) vs. 4.76 (2.45–7.89) %, p = 0.015] and a reduction in the ammonia serum level [59.50 (50.25–100) vs. 98 (73.75–138.25) mcg/dL, p = 0.010].

## Discussion

MHE is a complication of liver cirrhosis resulting from concomitant metabolic and hemodynamic alterations, which is often difficult to be diagnosed. Our study demonstrates that MHE is associated with alterations of cerebral vascular resistance, and that TCD represents a useful tool in the diagnosis of this complication of liver cirrhosis.

We observed an increase in MCA-RI and MCA-PI in cirrhotic patients that was significantly higher in the subgroup with MHE, similarly to what was previously observed in patients with overt hepatic encephalopathy^10,13,14^. Our study was also the first to assess the RI and PI of the PCA, that were both increased in patients with liver cirrhosis compared to controls, although they were not in patients with MHE compared to those without. Therefore, we concluded that MCA-RI and MCA-PI were the most reliable Doppler US parameters suggesting the occurrence of cerebral arterial flow abnormalities in patients with MHE.

To date, the mechanism by which cerebral vascular resistance increases in patients with hepatic encephalopathy has not yet been fully elucidated. The most probable hypothesis concerns a reduced cerebral metabolic activity, and the vasoconstriction of the brain arterial vessels related to the activation of the renin-angiotensin-aldosterone system and of the sympathetic nervous system, secondary to the splanchnic and systemic arterial vasodilation^20–23^. Indeed, it has been reported that cerebral vasoconstriction and the increase in MCA-RI are related to the increase in RA-RI^24^, suggesting a shared mechanism for vasoconstriction in these two districts. Accordingly, in our study population, we observed a significant correlation between RA-RI, SA-RI and MCA-RI, MCA-PI, PCA-RI and PCA-PI; however, despite the patients with MHE showed higher cerebral vascular resistance parameters compared to those without MHE, this was not true for the renal and splenic arteries. It is possible that, although cerebral and splanchnic arteries share the same mechanism of vasoconstriction related to the hyperdynamic circulation of liver cirrhosis, the development of the cognitive abnormalities typical of MHE could be due to other local or systemic factors, mainly affecting the intracranial vessels. Indeed, it should be noted that most of the patients included in the study presented an early stage liver disease, which often is not associated with significant portal hypertension; neuroinflammation derived by the gut microbiota in these patients may be advocated as an adjunctive mechanism not only in the development of MHE, but also in increasing cerebral vascular resistance through vasoactive effects^25^. This suggests that the evaluation of cerebral vascular resistance could have a relevant clinical impact in patients with liver cirrhosis, as TCD can detect changes occurring in the earliest stages of the cognitive impairment. Indeed, when TCD diagnostic performance was tested, we found that a MCA-RI cut-off of 0.65 had a 74% accuracy in the identification of patients with MHE, with a good sensitivity and specificity. TCD is therefore a promising tool for diagnosing and monitoring patients with MHE.

Ammonia is another factor playing an important role in the regulation of cerebral hemodynamics in patients with hepatic encephalopathy. Indeed, human studies have shown that ammonia is correlated with cerebral vascular resistance and that intravenous ammonia infusion in animal models causes the disappearance of cerebral flow self-regulation, and an increase in intracranial pressure and cerebral edema, which, in turn, can contribute to the increase in cerebral vascular resistance^26,27^. However, we did not find an association between ammonia serum levels and cerebral vascular resistance, although cirrhotic patients with MHE showed higher ammonia serum levels than patients without MHE. It is interesting to point out that cirrhotic patients with MHE also showed significantly higher MPV values compared to patients without MHE. We speculate that this could be related to a higher prevalence of occult intrahepatic porto-systemic venous shunts which could be responsible of both increased MPV (decreasing the intrahepatic outflow resistances) and serum ammonia level (causing a reduction in the detoxifying function of the liver).

This study is the first to explore changes in cerebral vascular resistance in patients with MHE after rifaximin therapy. MCA-RI and PI and PCA-RI and PI were not affected by rifaximin treatment at the dose of 400 mg tid for 15 days. Similarly, Kimer *et al*. failed to prove any significant influence of rifaximin over placebo on the hepatic venous pressure gradient (HVPG), systemic hemodynamics and MHE status in patients with cirrhosis and ascites^28^. In contrast, a recent study^29^ showed that cerebral vascular resistance decreases considerably after liver transplantation, as a consequence of the improvement of the inflammatory and hyperdynamic condition related to liver impairment. This probably means that only interventions able to modify the hemodynamic derangement of decompensated cirrhotic patients in a more profound and persistent manner, such as liver transplantation, allow to achieve a significant beneficial effect on cerebral vascular flow alterations.

The final objective of our study was to assess the endothelial function of patients with MHE and if it could be modified by rifaximin administration. Previous studies investigating FMD in patients with liver cirrhosis reported conflicting results^30–32^. We found that cirrhotic patients had an impaired endothelial function compared to controls, expressed by a lower FMD, reasonably as a consequence the hyperdynamic syndrome and systemic inflammation. The presence of MHE was not associated with a worse FMD in our study, confirming that liver cirrhosis is the main factor influencing the development of endothelial dysfunction. However, it is important to point out that we observed an improvement of FMD after rifaximin therapy in patients with MHE. This could be associated with its anti-inflammatory action secondary to the modulation of the gut microbiota, which is involved in the promotion, worsening and perpetuation of the hyperdynamic circulation and the inflammatory status of cirrhotic patients^33,34^.

In conclusion, compared to their counterpart without MHE, cirrhotic patients with MHE have significantly increased MCA-RI and MCA-PI, which cannot be improved by rifaximin treatment. A MCA-RI cut-off of 0.65 was able to distinguish with a good accuracy patients with MHE from those without, being therefore a promising diagnostic tool to be used in clinical practice.
